# Nutritional Status Is Associated With Survival Following Spinal Surgery in Patients With Metastatic Spinal Tumors

**DOI:** 10.7759/cureus.40451

**Published:** 2023-06-15

**Authors:** Masahiro Iinuma, Tsutomu Akazawa, Yoshiaki Torii, Jun Ueno, Shingo Kuroya, Atsuhiro Yoshida, Ken Tomochika, Takahiro Hideshima, Naoki Haraguchi, Hisateru Niki

**Affiliations:** 1 Department of Orthopaedic Surgery, St. Marianna University, Yokohama Seibu Hospital, Yokohama, JPN; 2 Department of Orthopaedic Surgery, St. Marianna University, Kawasaki, JPN; 3 Department of Orthopaedic Surgery, St. Marianna University School of Medicine, Kawasaki, JPN

**Keywords:** patient survival, nutritional status, prognostic scoring system, surgical treatment, metastatic spinal tumor

## Abstract

Background

Preoperative and postoperative nutritional statuses are reported to influence the outcomes and complications of multidisciplinary treatment, including patient survival. However, a causal relationship between nutritional status and survival following spinal surgery has not been demonstrated in patients with metastatic spinal tumors. The present study was, therefore, designed to evaluate the correlation between the nutritional status and survival following spinal surgery in patients with metastatic spinal tumors.

Methods

Nutritional status was evaluated using the Japanese version of the modified Glasgow prognostic score (JmGPS), C-reactive protein-to-albumin ratio (CAR), prognostic nutrition index (PNI), neutrophil-to-lymphocyte ratio (NLR), and platelet-to-lymphocyte ratio (PLR), which were calculated from the results of preoperative laboratory tests. The survival period was defined as the interval between the day preoperative data were obtained and the day of death.

Results

Data from 57 of 113 consecutive surgeries were retrieved. The CAR, JmGPS, and PNI were significantly correlated with the survival period (CAR, r = −0.576, P < 0.01; JmGPS, r = −0.537, P < 0.01; PNI, r = 0.316, P = 0.02). Furthermore, patients with 0 points on the JmGPS had significantly longer survival. Using receiver operating characteristic curves, CAR cutoffs of ≥0.880 and ≤0.220 were found to be optimal in predicting the 90- and 180-day postoperative survival, respectively.

Conclusions

The findings of the present study indicate that preoperative assessment of the JmGPS, CAR, and PNI has utility in estimating nutritional status and predicting survival following spinal surgery in patients with metastatic spinal tumors.

## Introduction

Surgery is commonly indicated for metastatic spinal tumors aimed at improving the quality of life and performance status of the patient. To achieve this goal, the accurate determination of prognosis in this patient population can aid surgeons when deciding on surgical indications. Accordingly, numerous prognostic scoring systems have been developed as tools for evaluating the prognosis of patients with metastatic spinal tumors [1,2]. Recently, the prevalence of bone metastasis has increased with the increasing number of patients with cancer, thereby leading to increased overall survival among patients with bone metastasis [3,4]. As a result, an increasing number of patients may benefit from surgical treatments for metastatic spinal tumors.

Preoperative and postoperative nutritional status reportedly influences the outcomes and complications of multidisciplinary treatment, including patient survival [5]. Nutritional status can be calculated using the following indices: the Japanese version of the modified Glasgow prognostic score (JmGPS) [6]; C-reactive protein (CRP)-to-albumin ratio (CAR) [7] calculated using serum CRP and albumin; prognostic nutrition index (PNI) [8,9] determined using serum albumin and total lymphocyte count (TLC); neutrophil-to-lymphocyte ratio (NLR) [10]; and platelet-to-lymphocyte ratio (PLR) [11].

Several studies have examined the relationship between nutritional status and clinical outcomes in nonmalignant diseases, including chronic obstructive pulmonary disease [12] and acute heart failure [13]. However, there is a lack of studies examining the relationship between nutritional status and clinical outcomes in patients with metastatic spinal tumors. Accordingly, the present study was designed to investigate the association between nutritional status and survival following spinal surgery in patients with metastatic spinal tumors.

## Materials and methods

Data collection

This retrospective study was approved by the Institutional Review Board of St. Marianna University School of Medicine with approval number 5713. The patients and/or their families were informed that data from the research would be submitted for publication and they gave their consent. From May 2009 to July 2021, 113 consecutive surgeries for metastatic spinal tumors were performed at our institution. Of the 113 surgeries, only 57 patients with complete data were included in the study analysis. Patients meeting any of the following criteria were excluded from the present study: alive at the end of the study period, unknown outcome (not known whether alive or dead), insufficient laboratory data, multiple myeloma or malignant lymphoma as the primary tumor, or biopsy of tumor tissues.

Indices for evaluating nutritional status

Nutritional status was evaluated using preoperative laboratory data. Laboratory tests were performed 12.6 ± 17.5 (mean ± SD) days before surgery. The survival period was defined as the interval between the day preoperative data were obtained and the day of death. All patients reportedly died due to the progression of the primary cancer.

JmGPS scores were calculated as follows: 0 points, CRP ≤ 0.5 mg/dL and albumin ≥ 3.5 g/dL; 1 point, CRP > 0.5 mg/dL or albumin < 3.5 g/dL; 2 points, CRP > 0.5 mg/dL and albumin < 3.5 g/dL (Table 1) [5,6].

**Table 1:** The contents of the Japanese version of the modified Glasgow prognostic scale Source: [6].

0	CRP ≤ 0.5 mg/dL and albumin ≥ 3.5 g/dL
1	CRP ≤ 0.5 mg/dL or albumin ≥ 3.5 g/dL
2	CRP > 0.5 mg/dL and albumin < 3.5 g/dL

The CAR was calculated by dividing serum CRP (mg/dL) by serum albumin (g/dL) [7]. The PNI was calculated using the following formula: 10 × albumin (mg/dL) + 0.005 × TLC (/µL) [9]. Furthermore, the NLR was calculated by dividing neutrophil count (/µL) by TLC (/µL) [10]. The PLR was calculated by dividing platelet count (/µL) by TLC (/µL) [11].

Surgical treatments

Surgeries were performed on patients who had neurological deficits or uncontrollable pain despite sufficient analgesic doses (including medical narcotics) [14]. It was also ensured that the patients and their families understood the risks of surgery. The surgical procedures included posterior decompression in five patients, posterior fixation in 46 patients (including 42 patients with decompression), anterior fixation in one patient, anteroposterior fixation in four patients (including one patient with decompression), and balloon kyphoplasty in one patient. Surgeries were performed on the cervical spine in 15 patients, the thoracic spine in 28 patients, and the lumbosacral spine in 14 patients.

Data analysis

All statistical analyses were performed using EZR (Saitama Medical Center, Jichi Medical University, Saitama, Japan), which is a graphical user interface for R (The R Foundation for Statistical Computing, Vienna, Austria). R is a modified version of R Commander designed to add statistical functions frequently used in biostatistics.

The correlation between nutritional status and survival period was evaluated using Spearman’s rank correlation coefficient test. Factors found to be correlated with survival were further evaluated using the Kaplan-Meier method, log-rank test, and receiver operating characteristic (ROC) curves. P-values <0.05 were considered statistically significant.

## Results

Data were collected in April 2022. Data were obtained from the case notes of 57 patients (32 males and 25 females; mean age 63.3 ± 11.9 years at the time of surgery). Primary lesions are shown in Table 2.

**Table 2:** Primary lesions

Primary lesion	No. of cases
Breast cancer	13
Lung cancer	11
Hepatocellular carcinoma	6
Renal cell carcinoma	6
Gastric cancer	4
Bile duct cancer	3
Prostate cancer	3
Colon cancer	2
Pancreatic cancer	2
Thyroid cancer	2
Malignant melanoma	1
Duodenal cancer	1
Ureteral cancer	1
Bladder cancer	1
Unknown	1

Major recorded complications included surgical site infection requiring debridement (four cases), postoperative hematoma (two cases, one patient required additional surgery), perioperative delirium (two cases), and surgical wound dehiscence (one case). Thirty-five patients underwent postoperative therapy for primary cancer (with 22 patients receiving molecular-targeted drugs and two patients receiving immune checkpoint inhibitors). Bone-modifying agents were used in 41 patients (19 patients treated with zoledronic acid and 22 patients treated with denosumab). The median survival period was 175.5 days (range, 28-2,393 days).

Correlation between nutritional status and survival period

**Figure 1:**
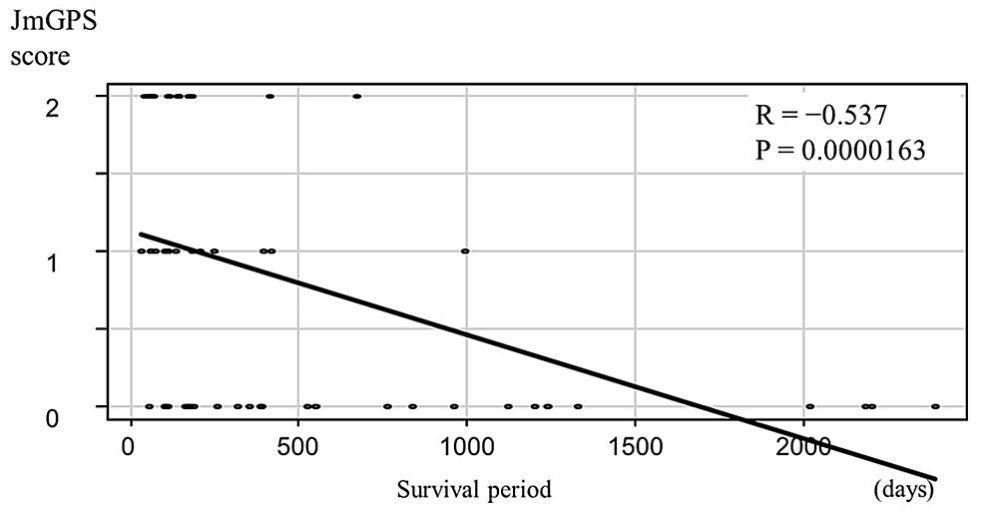
Correlation between the JmGPS and the survival period JmGPS scores were negatively correlated with the survival period. JmGPS, Japanese version of the modified Glasgow prognostic score.

The number of patients with 0, 1, and 2 points on the JmGPS was 26, 15, and 16, respectively. Survival period and JmGPS scores were significantly and negatively correlated (r = −0.537, P < 0.01; Figure 1).

**Figure 2:**
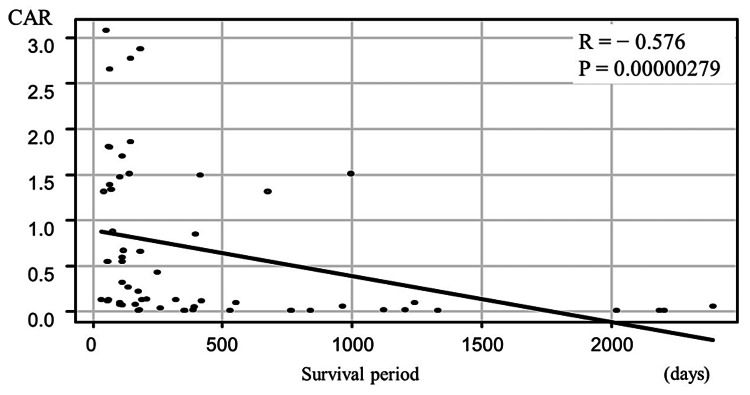
Correlation between the CAR and the survival period The CAR was negatively associated with the survival period. CAR, C-reactive protein-to-albumin ratio.

The mean CAR was 0.66 ± 0.84, which was significantly and negatively correlated with the survival period (r = −0.576, P < 0.01; Figure 2).

**Figure 3:**
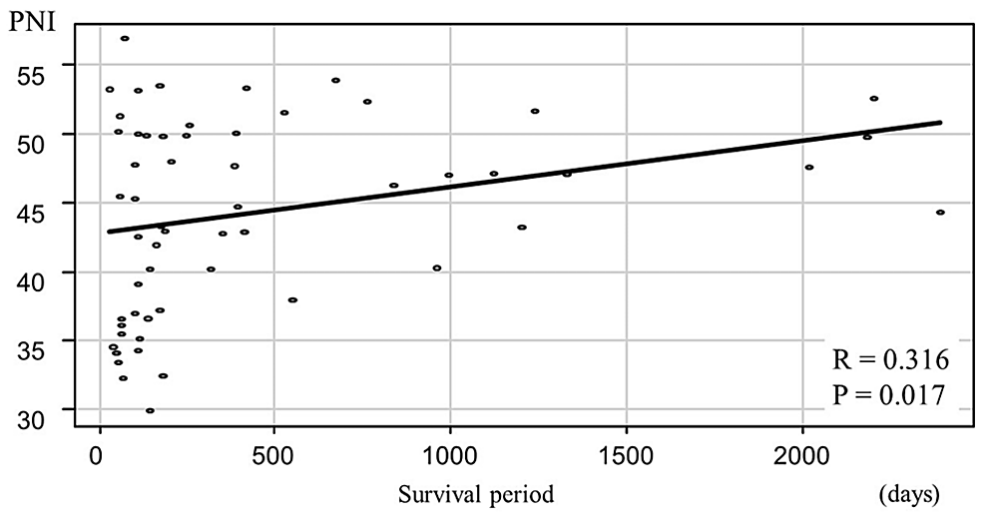
Correlation between the PNI and the survival period The PNI was positively correlated with the survival period. PNI, prognostic nutrition index.

Similarly, the mean PNI was 44.3 ± 6.89, which was significantly and positively correlated with the survival period (r = 0.316, P = 0.02; Figure 3).

**Figure 4:**
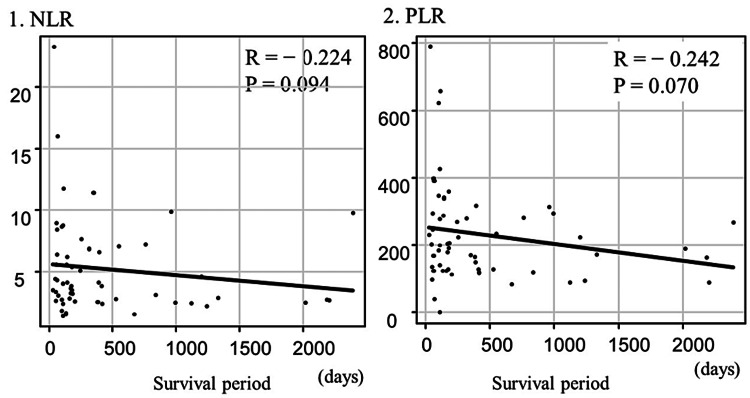
(1) Correlation between the NLR and the survival period. No correlation was observed between the NLR and the survival period. (2) Correlation between the PLR and the survival period. No correlation was observed between the PLR and the survival period NLR, neutrophil-to-lymphocyte ratio; PLR, platelet-to-lymphocyte ratio.

The mean NLR was 5.18 ± 3.82 and was not significantly correlated with the survival period (r = −0.224, P = 0.09). The mean PLR was 230.5 ± 143.6 and was not significantly correlated with the survival period (r = −0.242, P = 0.07; Figure 4).

Utility of nutritional status in predicting survival

**Figure 5:**
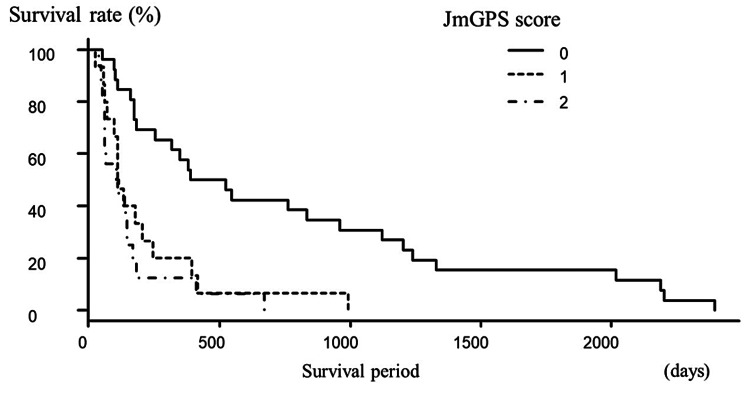
Survival according to JmGPS A significant difference in survival was observed between groups. JmGPS, Japanese version of the modified Glasgow prognostic score.

The above results demonstrate that the JmGPS, CAR, and PNI are significantly correlated with survival after spinal surgery for metastatic spinal tumors. The median survival period in patients with JmGPS scores of 0, 1, and 2 was 525 days (range, 55-239 days), 123.5 days (range, 28-993 days), and 112 days (range, 38-675 days), respectively. Significant differences were observed between the three groups using the Kaplan-Meier method and log-rank test (P < 0.01; Figure 5).

Using ROC curves, a CAR cutoff value of ≥0.88 was determined to be optimal in predicting survival at three months after surgery (sensitivity, 66.7%; specificity, 80.0%; area under the ROC curve [AUC], 0.783). Meanwhile, a CAR cutoff value of ≤0.220 was determined to be optimal in predicting survival six months after surgery (sensitivity, 69.0%; specificity, 75.0%; AUC, 0.766).

Using ROC curves, a PNI cutoff value of ≤36.5 was determined to be optimal in predicting survival at three months after surgery (sensitivity, 58.3%; specificity, 91.1%, AUC, 0.626). A PNI cutoff value of ≥42.5 was determined to be optimal in predicting survival at six months after surgery (sensitivity, 58.6%; specificity, 85.7%; AUC, 0.683).

## Discussion

The overall aim of the present study was to determine the correlation between nutritional statuses evaluated using indices based on laboratory tests and survival after spinal surgery in patients with metastatic spinal tumors. The findings of the present study demonstrate that the JmGPS, CAR, and PNI were significantly correlated with survival after spinal surgery in our study cohort. These findings imply that lower JmGPS and CAR scores and a higher PNI are associated with a longer duration of survival following spinal surgery. Patients with a JmGPS score of 0 points had a median survival period of one year or more; however, the survival period in patients with a JmGPS score of 1 or 2 points was several months. The CAR and PNI had utility in predicting three- and six-month survival in the present study.

Tokuhashi et al. [15] reported that an accurate evaluation of metastasis and prognosis is essential for selecting a suitable therapeutic strategy for metastatic spine tumors, owing to the limitations in treatment options. Numerous prognostic scoring systems have been developed [1,2], and their usefulness has been reverified [16-18]. However, recently, developments in cancer therapy have rapidly diversified, and the prognoses of patients with cancer have prolonged. Several studies reported that the prognosis based on a simple application of prognostic scoring systems has been underestimated [18,19]. Recently, accumulating evidence has clarified the role of inflammation-based prognostic scores, such as the JmGPS, CAR, PNI, NLR, and PLR [6-11].

Several studies have demonstrated the importance of evaluating nutritional status when managing patients with cancer. The accuracy of the JmGPS in predicting prognosis has been reported in patients with colorectal cancer [6] and hepatocellular carcinoma [20]. Furthermore, the accuracy of the CAR in predicting prognosis has been evaluated in patients with esophageal squamous cell carcinoma [21], breast cancer [22], and lung cancer [23]. Similarly, the accuracy of the PNI in predicting prognosis has been reported for gastric cancer [24], hepatocellular carcinoma [25], and pancreatic carcinoma [26]. Xu et al. [21] reported that the CAR has greater accuracy in predicting 12-, 24-, and 36-month survival compared to the NLR and GPS in patients with operable esophageal squamous cell carcinoma. However, few studies have evaluated the relationship between nutritional status and prognosis in patients with metastatic spinal tumors. The results of the present study demonstrate that the JmGPS, CAR, and PNI are good predictors of survival. Moreover, these nutritional status indices are based on serological tests, including CRP, albumin, and blood counts, which are frequently examined in clinical practice, thereby allowing nutritional status to be easily assessed.

The host-tumor interaction seriously influences the general condition of patients with cancer, including nutritional status and immune responses. One of these host-tumor interactions is cancer-induced weight loss, including cancer cachexia, which is an irreversible nutritional disorder. Cancer cachexia is a multifactorial syndrome defined by an ongoing loss of skeletal muscle mass and may lead to progressive functional impairment [27]. The American Society of Clinical Oncology guidelines states that cancer cachexia leads to fatigue, functional impairment, increased treatment-related toxicity, poor quality of life, and reduced survival [28]. A common measure of cancer cachexia is serological testing to determine nutritional status. Nutritional disorders reportedly occur at a relatively early stage of cancer and act as a prognostic factor independent of pathological factors [5].

The present study had some limitations. First, this was a single-institution study. Accordingly, bias may have been introduced into the study analysis due to differences in surgical indication, primary cancer site, and treatment strategy between patients. Second, this study only included patients who had undergone surgery. Patients receiving conservative treatment were not included due to the extremely short predicted survival duration, which may have influenced the results of our study. Third, the primary cancer site and the histological type of primary cancer were not compared due to the small sample size. Fourth, preoperative comorbidities, which may influence survival, were not evaluated. Finally, this was a retrospective study and postoperative treatment protocols varied between cases.

## Conclusions

Preoperative and postoperative nutritional statuses are reported to influence patient survival. However, a causal relationship between nutritional status and survival following spinal surgery has not been demonstrated in patients with metastatic spinal tumors. We have investigated the relationship between nutritional status and survival following spinal surgery in 57 patients with metastatic spinal tumors. Nutritional status was evaluated using the JmGPS, CAR, PNI, NLR, and PLR. In this study, JmGPS, CAR, and PNI showed a significant correlation with the survival period. Also, CAR and PNI were useful to predict 90- and 180-day postoperative survival, respectively. Although the nutritional status was easily calculated from blood samples, it is a useful index that can be used to predict the survival of patients with metastatic spinal tumors.
